# Baseline characteristics influencing quality of life in women undergoing gynecologic oncology surgery

**DOI:** 10.1186/1477-7525-5-25

**Published:** 2007-05-17

**Authors:** Karen M Gil, Heidi E Gibbons, Eric L Jenison, Michael P Hopkins, Vivian E von Gruenigen

**Affiliations:** 1 Department of Obstetrics and Gynecology, Akron General Medical Center, Akron, Ohio, 44302, USA; 2 Northeastern Ohio Universities College of Medicine, Rootstown, Ohio, 44272, USA; 3 Department of Obstetrics and Gynecology, University Hospitals Case Medical Center, Case Western Reserve University, Cleveland, Ohio, 44106, USA

## Abstract

**Background:**

Quality of life (QoL) measurements are important in evaluating cancer treatment outcomes. Factors other than cancer and its treatment may have significant effects on QoL and affect assessment of treatments. Baseline data from longitudinal studies of women with endometrial or ovarian cancer or adnexal mass determined at surgery to be benign were analyzed to determine the degree to which QoL is affected by baseline differences in demographic variables and health.

**Methods:**

This study examined the effect of independent variables on domains of the Functional Assessment of Cancer Therapy (FACT-G) pre-operatively in gynecologic oncology patients undergoing surgery for pelvic mass suspected to be malignant or endometrial cancer. Patients also completed the Short Form Medical Outcomes Survey (SF-36) questionnaire (a generic health questionnaire that measures physical and mental health). Independent variables were surgical diagnosis (ovarian or endometrial cancer, benign mass), age, body mass index (BMI), educational level, marital status, smoking status, physical (PCS) and mental (MCS) summary scores of the SF-36. Multiple regression analysis was used to determine the influence of these variables on FACT-G domain scores (physical, functional, social and emotional well-being).

**Results:**

Data were collected on 157 women at their pre-operative visit (33 ovarian cancer, 45 endometrial cancer, 79 determined at surgery to be benign). Mean scores on the FACT-G subscales and SF-36 summary scores did not differ as a function of surgical diagnosis. PCS, MCS, age, and educational level were positively correlated with physical well-being, while increasing BMI was negatively correlated. Functional well-being was positively correlated with PCS and MCS and negatively correlated with BMI. Social well-being was positively correlated with MCS and negatively correlated with BMI and educational level. PCS, MCS and age were positively correlated with emotional well-being. Models that included PCS and MCS accounted for 30 to 44% of the variability in baseline physical, emotional, and functional well-being on the FACT-G.

**Conclusion:**

At the time of diagnosis and treatment, patients' QoL is affected by inherent characteristics. Assessment of treatment outcome should take into account the effect of these independent variables. As treatment options become more complex, these variables are likely to be of increasing importance in evaluating treatment effects on QoL.

## Background

Women diagnosed with gynecologic cancer are at risk for depression, anxiety and reduced quality of life (QoL) [1-4]. QoL is an important component of assessing the effects of surgery, radiation, and chemotherapy [5]. In addition to clinical variables, QoL in cancer patients undergoing treatment is affected by demographic variables, socio-economic status, social characteristics and personal expectations [6,7]. Pretreatment factors have been found to influence QoL in patients undergoing radiation therapy [8]. Significant differences in QoL were found as a function of age, race, Karnofsky Performance Status (KPS), income level and employment status [8]. Pretreatment Functional Assessment of Cancer Therapy (FACT-G) scores were higher in patients who were older, white, had higher KPS scores, were married, had a higher income and were college graduates. Gender and primary site of disease did not have an effect. Arredondo et al. examined QoL in men with prostate cancer and found men with more comorbidities had significantly worse scores at baseline in the physical domains [9].

Pretreatment characteristics may affect patients' reaction to their illness and treatment and thus influence disease specific QoL scores measured during treatment. The role these baseline characteristics play in women's ability to maintain good QoL following diagnosis and during treatment may therefore affect assessment of treatment and become a factor in determining which treatments are selected. A health status questionnaire was used to capture the effect of general physical and mental health as extensive personal information was not available on these women. The SF-36 was selected as it provides a measure of the health burden of chronic disease and other medical conditions that the women may have [10]. Baseline, pre-operative, data from longitudinal studies of women with endometrial or ovarian cancer [11,12] were analyzed to determine the degree to which QoL, measured with a disease specific questionnaire, is affected by baseline differences in demographic variables, and physical and mental health measured with the SF-36. At the time these data were obtained, women were unaware of their ultimate diagnosis and/or stage of disease. Women with an adnexal mass determined at surgery to be benign were included to control for the effect of cancer.

## Methods

This prospective study was conducted at two gynecologic oncology offices located in Northeastern Ohio. Consecutive patients requiring surgery for a pelvic mass or a positive endometrial biopsy (endometrial cancer) were enrolled in a longitudinal assessment of QoL study at their pre-operative visit between January 2001 and July 2004.

Questionnaires (described below) were completed at the initial office visit following informed consent to participate in this IRB approved study. Baseline demographics were ascertained by interview with a research assistant. Private office records were reviewed to obtain height, weight and diagnosis following surgery. Body mass index (BMI) was calculated (defined as weight (kg) divided by height (m^2^) for each woman) and categorized as normal weight (18.5–24.9), overweight (25–29.9) or obese (BMI ≥ 30) [13]. Smoking was quantified in pack years, a method used to measure the amount a person has smoked over a long period of time. It is calculated by multiplying the number of packs of cigarettes smoked per day by the number of years the person has smoked [14].

General health status was measured with the Short Form Medical Outcomes Survey (SF-36), a comprehensive survey designed to measure physical and mental health [10]. There are 8 subscales, summarized into physical and mental composite scores. Subjects are asked if their health limits activities during a regular day (from vigorous activities to bathing or dressing), if they have problems with work or other activities due to physical health or emotional problems, how they have been feeling emotionally during the last 4 weeks, if they have pain and how they rate their health. The SF-36 is a widely used, reliable and validated instrument with population specific norms that can be used to evaluate the burden of different diseases and treatments. The questionnaire was used in this study to assess patients' baseline level of physical and mental health.

QoL was measured with the Functional Assessment of Cancer Therapy (FACT-G), a 27 item core questionnaire evaluating physical, functional, social and emotional well-being within the previous 7 days [15]. While some questions are similar to those asked on the SF-36, most questions are specific to cancer and its treatment. For example, both instruments ask if the patient has pain, and if they are full of pep (SF-36) or have a lack of energy (FACT-G), however, the FACT-G was developed specifically to include items that are affected by the diagnosis of cancer and its treatment. Items are summed to give scores for each domain. All domains are scored so that a higher score indicates higher QoL [16]. The FACT-G is a reliable and validated instrument for measuring QoL in cancer patients, including the elderly [15,16].

In addition to analyzing the four domain scores, five questions were selected a priori for analysis to determine the distribution of responses (not at all to very much). Two questions not directly related to treatment were selected from the physical well-being domain, and one question each from the social, emotional and functional well-being domains. The questions addressed level of energy, pain, support from family, feeling nervous and ability to enjoy life. Prior research in cancer patients has indicated these areas as problematic [2]. Responses to these questions were analyzed to determine how many women were already experiencing difficulties in these clinically relevant areas that are likely to become increasingly important during the treatment process.

In this study, touch screen computers with 15 inch monitors were programmed with the FACT-G questionnaire [17]. Sequential screens are programmed with one domain per screen; patients complete the questionnaire by touching responses to each question within the domain. The physical, social, and functional well-being screens have 7 questions on the screen; the emotional well being screen has 6 questions. Patients may change their answer by touching an alternate response on that screen but cannot return to a previous screen. Patients completed the questionnaire on their own, but the research assistant was available if they had questions. Patients were given the option of completing the paper version of the questionnaire, but used the computer the majority of the time [17]. The effect of using the computer, versus completing the questionnaires on paper, has been previously described [17]. Women were found to respond to the content of the questionnaires and not the method of administration.

### Statistical analysis

#### Mean QoL (FACT-G) and general health status (SF-36) scores

Scores on the FACT-G subscales and the SF-36 summary scores were analyzed as a function of diagnosis (ovarian or endometrial cancer, benign adnexal mass). Outcomes were not analyzed as a function of stage of disease as the majority of ovarian cancer patients were stage III/IV and nearly all endometrial cancer patients were Stage I/II. Scores on the FACT-G subscales and SF-36 summary scores were compared with normative data. Differences greater than 2–3 points were considered clinically important for the FACT-G physical and functional subscales and greater than 2 points for the FACT-G emotional subscale [16]. Analysis of variance was used to determine if there were significant differences as a function of diagnosis.

#### Univariate Analyses

Age, BMI, education and number of pack years smoked were analyzed as continuous variables and marital status and diagnosis were analyzed as categorical variables. Marital status was categorized as being married or non-married (single, divorced, and widowed) and differences were analyzed with the t-test. Pearson correlation coefficients were calculated for normally distributed continuous variables (age, BMI), SF-36 physical summary score (PCS) and mental summary score (MCS) with FACT-G domains. Spearman's correlation coefficients were calculated for non normally distributed continuous variables (education, pack years smoked) with FACT-G domains.

**Multiple regression **analysis was used to assess the effect of PCS and MCS scores, diagnosis, age, BMI, educational level, marital status, and smoking status on FACT-G domain scores. For this analysis, educational level was categorized as < 16 years versus ≥ 16 years (college degree or higher) and smoking was categorized according to < 5.0 versus ≥ 5.0 pack years. The variables were regressed on individual FACT-G domains (physical, functional, emotional and social) using a stepwise linear regression procedure. Variables were entered in the regression models at a probability level of p < 0.05 and were removed at a probability level > 0.10. Collinearity diagnostics (tolerance and variance inflation factor) were examined to assess multi-collinearity of variables included in the final model. SPSS version 14.0 (Chicago, IL) was used for analysis.

## Results

Of the 212 consecutively approached patients, 172 agreed to participate (81%). Only one of the two questionnaires was completed by 15 patients and they were excluded from subsequent analysis (final n = 157).

Fifty percent of the women had a diagnosis of cancer; 73% were overweight, 36% smoked at some time point, 35% were college graduates and most were married (Table 1). The majority of women with ovarian cancer were diagnosed with Stage III/IV disease; nearly all women with endometrial cancer had Stage I or II disease.

**Table 1:** Patient demographics, #(%)

Age, mean (SD), range	58.76 (13.6), 26–86 years
	
Final diagnosis	
Ovarian cancer	33 (21%)
Endometrial cancer	45 (29%)
Benign adnexal mass	79 (50%)
	
Overweight or obese (BMI ≥ 25)	
Ovarian cancer	22 (67%)
Endometrial cancer	38 (84%)
Benign adnexal mass	54 (68%)
	
Smoking	
Never	101 (64%)
< 5.0 pack years	10 (6%)
> 5.0 pack years	46 (30%)
	
College graduate	55 (35%)
	
Stage of cancer	
Ovarian cancer	
I/II	12 (36%)
III/IV	21 (64%)
	
Endometrial cancer	
I/II	43 (96%)
III/IV	2 (4%)
	
Married	91 (58%)

Scores from the FACT-G and the SF-36 are presented in Table 2. There were no significant differences for any of the FACT-G subscales or the SF-36 summary scores as a function of diagnosis (all F values less than 1.7; all p values > 0.19). Normative data for FACT-G subscales from women with cancer were similar to those from women in the general population with the exception of social well-being scores, which were slightly higher in women with cancer [18]. Scores for patients with ovarian cancer in this study were within 2 points of normative data for cancer patients on all the FACT-G subscales. Scores for patients with endometrial cancer differed by less than 3 points on the physical well-being subscale and differed by 2 points or less for the other subscales of the FACT-G relative to normative data for cancer patients. Physical and functional well-being subscale scores for women with a mass determined at surgery to be benign were within 2 points of normative data for women with cancer and women in the general population. Mean score on the social well-being subscale of the FACT-G was slightly higher (2.4 points) than normative data from women in the general population but the same as normative data from women with cancer. Mean score on the emotional well-being subscale was slightly lower (2.4 points) than normative data for women in the general population, but within 2 points of normative data from women with cancer. Patients' physical and mental composite scores from the SF-36 were within 2 to 3 points of data collected in women from the US population aged 55–64 [19].

**Table 2:** Mean (SD) quality of life (FACT-G) and general health status (SF-36) scores

	Mean	SD	Normative Data – Cancer, Female (18)	Normative Data – General US Female (18)	Normative Data – Women Age 55–64 (19)
Physical, FACT-G (0–28)	23.3	4.6	21.60	22.10	
Ovarian cancer	23.3				
Endometrial cancer	24.0				
Benign adnexal mass	23.0				
					
Functional, FACT-G (0–28)	20.2	4.5	19.50	18.30	
Ovarian cancer	19.4				
Endometrial cancer	21.5				
Benign adnexal mass	19.7				
					
Social, FACT-G (0–28)	22.3	4.6	22.30	19.80	
Ovarian cancer	22.8				
Endometrial cancer	22.2				
Benign adnexal mass	22.2				
					
Emotional, FACT-G (0–24)	17.5	6.2	18.70	19.40	
Ovarian cancer	18.5				
Endometrial cancer	17.6				
Benign adnexal mass	17.0				
					
PCS, SF-36	43.6	11.7			45.03
Ovarian cancer	42.7				
Endometrial cancer	45.7				
Benign adnexal mass	42.7				
					
MCS, SF-36	49.4	10.6			50.56
Ovarian cancer	51.4				
Endometrial cancer	49.0				
Benign adnexal mass	48.8				

Correlations between the FACT-G domains and clinical and demographic variables are presented in Table 3. SF36 physical (PCS) and mental health (MCS) scores, age, and educational level were positively correlated with physical well-being while increasing BMI was negatively correlated. Functional well-being was positively correlated with PCS and MCS and negatively correlated with BMI. Social well-being was positively correlated with MCS, and negatively correlated with BMI and educational level. PCS, MCS and age were positively correlated with emotional well-being. There were no differences in mean scores on the FACT-G subscales between women who were married or not married and no significant correlations between number of pack years smoked and subscales on the FACT-G (data not shown).

**Table 3:** Univariate Analyses: Correlations between FACT domains, SF36 (PCS, MCS), demographic variables and smoking status

**FACT Domain**	**PCS**	**MCS**	**Age**	**BMI**	**Education**
Physical	.480***	.430***	.275***	-.191**	.191*
Functional	.372***	.445***	.125	-.154*	.119
Social	.103	.184*	.016	-.161*	-.168*
Emotional	.141*	.578***	.307***	-.128	.065

*p <.05, ** p < .01, *** p < .001

PCS and MCS accounted for a significant amount of the variance (R^2^) in regression models for each of the FACT-G domains (Table 4). Models that included physical and mental health accounted for a significant amount of the variability in well-being scores. The models accounted for 30 to 44% of the variability in baseline physical, emotional, and functional well-being.

**Table 4:** Multiple linear regression analysis for each FACT-G domain

**FACT domain**	**Variables**	**Standardized Beta coefficient**	**t value**	**p value**	**R, adjusted R2**	**F value, p value**
Physical	PCS	0.486	8.06	< 0.01	0.67, 0.44	41.9,
	MCS	0.351	5.69	<0.01		p < 0.001
	Age	0.244	3.94	<0.01		
						
Functional	MCS	0.428	6.44	<0.01	0.57, 0.31	36.4,
	PCS	0.351	5.27	<0.01		p < 0.001
						
Social	MCS	0.207	2.64	<0.01	0.31, 0.08	5.4,
	College Grad	-0.205	-2.61	0.010		p = 0.001
	BMI	-0.170	-2.19	0.030		
						
Emotional	MCS	0.525	8.04	<0.01	0.62, 0.37	31.8,
	Age	0.201	3.07	<0.01		p < 0.001
	PCS	0.135	2.11	0.037		
						
Total	MCS	0.493	7.96	<0.01	0.67, 0.44	41.01,
	PCS	0.368	6.06	<0.01		p < 0.001
	Age	0.158	2.55	0.01		

At baseline, there was a range of responses to questions that addressed issues such as level of energy, pain, emotional support from family, feeling nervous and being able to enjoy life (Table 5). Approximately half of the women had been feeling a little bit or somewhat nervous for the past 7 days (58%) and an additional 26% had been feeling quite a bit or very nervous. Half of the women responded a little bit or somewhat when asked if they had a lack of energy (55%) or pain (48%). Eighty percent of them responded they were able to enjoy life and received emotional support from their family quite a bit or very much.

**Table 5:** Individual FACT-G questions

	**Not at all**	**A little bit/Somewhat**	**Quite a bit/Very much**
"I have a lack of energy"	37 (24%)	87 (55%)	33 (21%)
"I have pain"	61 (39%)	75 (48%)	31 (20%)
"I get emotional support from my family"	9 (6%)	18 (12%)	130 (83%)
"I feel nervous"	25 (16%)	91 (58%)	41 (26%)
"I am able to enjoy life"	3 (2%)	28 (18%)	126 (80%)

## Discussion

At the time of this initial visit with a gynecologic oncologist, all women were aware that they would be undergoing major surgery, either for endometrial cancer or to remove a pelvic mass. At that time, women did not know their disease status and/or stage of disease. Mean QoL and general health scores of women were similar to those of population norms from the US general population or from cancer patients. Mean social well-being scores for all groups in this study were similar to population norms for cancer patients but higher than that for the general population. This difference has been observed before and may be due to women seeking social support during this time [18].

Despite the fact that mean scores were similar to reference standards, there was a range of scores on the QoL survey. The majority of the women were enjoying life, but approximately half were already reporting a little bit or somewhat lack of energy and pain. Women with better general physical and mental health had higher physical, functional and emotional well-being scores on a disease specific QoL instrument. Age was positively correlated with physical and emotional well-being in univariate and multivariate analyses. Wan et al examined the relationship between demographic variables (including age), clinical factors and social characteristics and measures on the four subscales of the FACT-G in cancer patients who had completed a minimum of two cycles of chemotherapy or 10 radiation therapy treatments [7]. They found lower QoL scores among those with poorer performance status, younger patients and lower SES. Consistent with their results, in this study younger age was associated with lower physical, emotional and total QoL scores. A recent study of 2208 women with breast cancer who completed the EORTC general cancer QoL scale (breast cancer module) found that younger age was a significant risk factor for poorer QoL [20]. Movsas et al. speculated that the observation of decreased QoL in younger patients may be due to the devastating impact of a cancer diagnosis at a younger age [8].

Higher BMI was negatively correlated with physical and functional well-being in univariate analyses. Obesity has not specifically been linked to scores on the FACT-G, however, it has been linked to lower scores on the SF-36 [21]. In this study, increased BMI did not continue to be significant in multivariate analyses of physical and functional well-being when the SF-36 summary scores were included. BMI continued to be a significant independent variable included in the model for social well-being. Weight, BMI, waist and hip circumference and waist-hip-ratio are all strongly associated with increased risk of endometrial cancer [22], and obesity is increasing in the United States [23]. Decreased general health status and decreased QoL may become increasingly important in assessing treatment outcomes for women with endometrial cancer.

A model that included physical and mental health summary scores from the SF-36 and age accounted for 44% of the variability in the total FACT-G scores. This is similar to the findings by Wan et al. that 45% of the variability in total FACT-G scores in cancer patients who had undergone treatment was accounted for by demographic variables (age, gender, living arrangement, race/ethnicity, SES), clinical variables (performance status, disease type and stage) and social factors (spiritual beliefs, religious affiliation and relationship with physician) [7]. These data suggest that physical and mental health and age are able to account for a significant amount of the variability in pre-treatment QoL scores in women undergoing gynecologic surgery.

The main limitation of this study is the small sample size and the lack of diversity. The lack of diversity may have allowed the authors to determine the effect of the independent variables on the subscales of the FACT-G but also limits the generalizability of the results. These results should be replicated, and expanded, in larger samples of women.

FACT-G subscale scores did not differ significantly as a function of diagnostic group (Table 2) and diagnosis was not a significant variable in the multiple regression models. None of the women had started treatment for their illness and all were facing a similar first step. This reinforces the observation in this study that baseline characteristics unrelated to disease are already affecting QoL in areas that will eventually become important for women undergoing long-term treatment.

## Conclusion

Specific aspects of QoL that will be affected by cancer and treatment are affected by level of education, lifestyle and general health. Women, at baseline, have a range in level of physical and emotional health. These baseline characteristics may affect women's ability to tolerate surgery, regimens such as IP chemotherapy, consolidation chemotherapy and secondary surgical debulking. Further research should examine whether women beginning the therapeutic process at a disadvantage have difficulties completing therapy and whether early interventions increase later QoL.

## Competing interests

The author(s) declare that they have no competing interests.

## Authors' contributions

KG and VVG conceived of the study and design. VVG, HG, EJ and MH implemented the study and were responsible for day to day conduct of the study. KG and HG analyzed the data. KG, HG and VVG drafted the manuscript; EJ and MH provided critical review. All authors read and approved the final manuscript.
